# Cardiovascular risk profile of Middle Eastern immigrants living in the United States-the National Health Interview Survey

**DOI:** 10.1016/j.ajpc.2021.100312

**Published:** 2021-12-27

**Authors:** Tamer Yahya, Isaac Acquah, Mohamad B Taha, Javier Valero-Elizondo, Mouaz H. Al-Mallah, Mohammed A. Chamsi-Pasha, William A. Zoghbi, Ahmed Soliman, Nadeen Faza, Miguel Cainzos-Achirica, Khurram Nasir

**Affiliations:** a Division of Cardiovascular Prevention and Wellness, Department of Cardiology, Houston Methodist DeBakey Heart and Vascular Center, 6550 Fannin St Suite 1801, Houston TX 77030, USA; b Center for Outcomes Research, Houston Methodist, Houston TX, USA

**Keywords:** Cardiovascular disease, Epidemiology, Middle Eastern, Risk factors, ASCVD, atherosclerotic cardiovascular disease events, CI, confidence interval, CRF, cardiovascular risk factors, CVD, cardiovascular disease, ME, Middle Eastern, NHIS, National Health Interview Survey, NHW, non-Hispanic White

## Abstract

**Background:**

Middle Eastern (ME) immigrants are one of the fastest-growing groups in the US. Although ME countries have a high burden of atherosclerotic cardiovascular disease (ASCVD), the cardiovascular health status among ME immigrants in the US has not been studied in detail. This study aims to characterize the cardiovascular health status (CVD risk factors and ASCVD burden) among ME immigrants in the US.

**Methods:**

We used 2012–2018 data from the National Health Interview Survey, a US nationally representative survey. ME origin, CVD risk factors, and ASCVD status were self-reported. We compared these to US-born non-Hispanic white (NHW) individuals in the US.

**Results:**

Among 139,778 adults included, 886 (representing 1.3 million individuals, mean age 46.8) were of ME origin, and 138,892 were US-born NHWs (representing 150 million US adults, mean age 49.3). ME participants were more likely to have higher education, lower income and be uninsured. The age-adjusted prevalence of hypertension (22.4% vs 27.4%) and obesity (21.4% vs 31.4%) were significantly lower in ME vs NHW participants, respectively. There were no significant differences between the groups in the age-adjusted prevalence of ASCVD, diabetes, hyperlipidemia, and smoking. Only insufficient physical activity was higher among ME individuals. ME immigrants living in the US for 10 years or more reported higher age-adjusted prevalence of hypertension, hyperlipidemia, and ASCVD.

**Conclusions:**

ME immigrants in the US have lower odds of hypertension and obesity, and of having a suboptimal CRF profile compared to US-born NHWs. Further studies are needed to determine whether these findings are related to lower risk, selection of a healthier ME subgroup in NHIS, or possible under-detection of cardiovascular risk factors in ME immigrants living in the US.

## 1 Introduction

The Middle East refers to a large geographical area comprised of numerous countries in West Asia, Southeast Europe, the Arab peninsula, and a part of North Africa [1]. ME immigrants are a rapidly growing population in the US, accounting for approximately 3% of all immigrants [2]. The size of the ME immigrant population in the US has been estimated to be between 1.2–3.7 million [1,3], however, an accurate census is lacking due to the racialization of ME immigrants as “White” by the US census [1].

While the burden and risk of cardiovascular disease (CVD) in certain minority groups in the US has been well-described [4,5], it remains scarcely studied in others, including ME immigrants. Current literature on the cardiovascular health of ME immigrants in the US is limited and often combined with other immigrant groups [2,6]. Studies until 2016 have suggested that the prevalence of hypertension, obesity, and diabetes among ME immigrants in the US was unexceptional compared to other immigrant groups in the country [2]. However, ME immigrants have been noted to increasingly report a poor health status over the past two decades [3], and have low use of health care services [1]. Moreover, studies in ME countries have noted a high burden of CVD and its risk factors that is still rising [7,8]. These factors bring into question whether available prevalence estimates in US ME immigrants are truly representative of their cardiovascular risk profile and stress the need for updated analyses.

In this study, we used data from a nationally representative sample of the US to characterize the cardiovascular risk profile and the burden of atherosclerotic CVD (ASCVD) among ME immigrants, and explore the effects of social determinants and length of stay in the US on their cardiovascular health.

## 2 Methods

### 2.1 Setting and study design

This was a cross-sectional analysis using data from the 2012–2018 National Health Interview Survey (NHIS). We pooled data from years 2012–2018 to garner enough power in order to describe the attributes related to the ME population. Annually, the National Center for Health Statistics administers the survey to a nationally representative sample of civilian non‐institutionalized US individuals [9]. Out of the five core components of NHIS (the household file, the family file, the person, the sample adult file and the sample child file), we used the sample adult file combined with additional variables from other components [10]. The household file describes the characteristics of each household, while the family file describes the characteristics of the families living in those households. The person file variables are derived from the sections making up the family core of the NHIS which is then collected for household members. The sample adult and child sections cover many of the subject areas included in the family core with more specific and detailed information [10]. NHIS data is publicly available and de-identified data, therefore our study was exempt from the Institutional Review Board of Houston Methodist Hospital [11].

### 2.2 Study population and exposures

Our study population was restricted to NHIS participants ≥18 years of age. Two groups were included: participants who self-reported being born in the Middle East (“ME immigrants”, including naturalized citizens, legal permanent residents, refugees, undocumented immigrants, and individuals on visas, including students or guest workers [12], and people who self‐identified as non-Hispanic White (NHW) and self-reported being born in the US (“US-born NHWs”, which served as reference population for this analysis). The NHIS uses the CIA on-line *World Factbook*  to place countries into regional categories [13], in which the middle east is defined by the following countries: Armenia, Azerbaijan, Bahrain, Georgia, Iran, Iraq, Jordan, Kuwait, Lebanon, Oman, Qatar, Palestine (Gaza Strip and West Bank), Saudi Arabia, Syria, Turkey, United Arab Emirates, and Yemen [14].

### 2.3 Cardiovascular risk factors and ASCVD

The risk factors assessed in this analysis were all self-reported and included: hypertension, diabetes, high cholesterol, obesity (calculated body mass index ≥ 30 kg/m^2^), current smoker, or insufficient physical activity (based on not participating in > 150 min per week of moderate-intensity aerobic physical activity, > 75 min per week of vigorous-intensity aerobic physical activity, or a total combination of ≥ 150 min per week of moderate/vigorous-intensity aerobic physical activity). Based on the presence of these individual risk factors, individuals were also categorized as having an “optimal” (0–1 risk factors) or “suboptimal” (≥ 2 risk factors) cardiovascular risk factor (CRF) profile (Supplementary Table 1.).

In a similar fashion, ASCVD status was self-reported. Individuals were deemed to have ASCVD if they answered “yes’ to any of the following questions in NHIS: “*Have you ever been told by a doctor or other health professional that you had… Angina, also called angina pectoris? …Coronary heart disease? …A stroke? …A heart attack, also called myocardial infarction?”.*

### 2.4 Covariates

Other relevant variables in this study included age, sex, education, insurance status, family income, usual source of care, length of stay in the US, and English language proficiency. Categorical variables were classified as follows: age (18–44, 45–64 and ≥ 65), sex (male and female), education (some college or higher or high school or lower), insurance type (insured, and uninsured) family income (based on the percent of family income to the federal poverty limit from the Census Bureau: middle/high-income [≥ 200], low-income [< 200%]); English proficiency (speaks english well, and doesn't speak english well), and source of care (has a usual source of care, and doesn't have a usual source of care).

### 2.5 Statistical analyses

We used survey-specific descriptive statistics to obtain national estimates of cardiovascular risk factors in the study participants by study group (ME vs US-born NHW). The continuous age variable was reported as mean and standard deviation, and categorical variables were reported using counts and weighted percentages. We used chi-squared tests to compare frequencies between the two groups for categorical variables.

The NHIS is a multistage, complex probability sample which after reaching a given number of observations, can produce nation-level estimates. To achieve this, sampling weights, provided by NHIS, are used to calculate national (weighted) estimates from the unweighted observations using statistical software. We used the “svy” command to obtain weighted national estimates for the prevalence of ASCVD and risk factors by the region of birth (ME vs US-Born NHW) in addition to estimates in ME immigrants by the length of stay in the US. Similarly, we obtained weighted prevalence estimates for suboptimal CRF profile across socio-demographic characteristics. Age-adjusted estimates were obtained using the US Census Population 2010 Data [15].

Multivariable logistic regression models were used to analyze the association between ME origin (compared to US-born NHWs) and each individual cardiovascular risk factor, suboptimal CRF, and suboptimal CRF in those without ASCVD. Logistic regression models were progressively of adjusted for potential confounders as follows: Model 1 was unadjusted, Model 2 adjusted for age and sex, and Model 3 was further adjusted for income, insurance, education, and having a usual source of care.

Similarly, logistic regression models were used to estimate the associations between socio-demographic factors and suboptimal CRF profile in ME immigrants. Model 1 was unadjusted, and Model 2 was adjusted for all other socio-demographic characteristics.

We obtained variance estimations for the entire pooled cohort from the Integrated Public Use Microdata Series (http://www.ipums.org) [16]. The person-level NHIS sample weights (representing the inverse probability of a person being selected and household response adjustment) were divided by the number of years in the pooled datasets to accurately reflect the total population, as per NHIS standards [16]. For all statistical analyses, a two-tailed alpha level of 0.05 was considered statistically significant, and to account for the complex sampling design of the NHIS to estimate annual nationally representative data, we utilized the -svy- family of commands in Stata. All analyses were performed using Stata version 16 (StataCorp, College Station, TX).

## 3 Results

### 3.1 Study population

Our final study sample included 139,778 participants, representing approximately 151.7 million US adults annually. Of this number, 886 identified as ME immigrants (representing 1.31 million), and 138,892 (representing approximately 150.4 million) identified as NHW born in the US.

### 3.2 Sociodemographic characteristics

Compared to US-born NHW, ME immigrants were younger (mean age 49.3 vs 46.8), and more likely to be male (48.5% vs. 55%). ME immigrants were also more likely to have higher education, lower income, and were more frequently uninsured. They were also less likely to have a usual source of care (Table 1).

**Table 1:** Characteristics of the study population by region of birth, NHIS 2012–2018.

Variables	Middle Eastern born	US-born Non-Hispanic White	P-Value
Sample size (unweighted)	886	138,892	–
Sample size (weighted)	1309,955	150,351,487	
			
Age, mean (SD)	46.8 (14.7)	49.3 (18.3)	0.016
Age Category, n (%)			0.0016
18–44	443 (47.4)	50,330 (41.1)	
45–64	283 (36)	48,734 (36)	
65 & above	160 (16.3)	39,828 (22.9)	
Sex, n (%)			<0.001
Male	505 (55)	63,781 (48.5)	
Female	381 (45)	75,111 (51.5)	
Education, n (%)			<0.001
High school or less	230 (28.7)	46,908 (33.4)	
Some College or higher	649 (71.3)	91,629 (66.6)	
Income, n (%)			<0.001
Middle/High Income	466 (60.5)	91,825 (76.6)	
Low-Income	355 (39.5)	36,086 (23.4)	
Insurance, n (%)			0.001
Insured	758 (87.5)	125,889 (91.8)	
Uninsured	113 (12.5)	11,397 (8.2)	
Years in the US, n (%)			–
Less than 10 years	318 (33.9)	–	
More than 10 years	566 (66.1)	–	
Has Usual Source of Care, n (%)	705 (81.4)	122,238 (88.6)	<0.001


Abbreviations: NHIS, National Health Interview Survey; SD, standard deviation; US, United States.

### 3.3 Age-adjusted burden of cardiovascular risk factors and ASCVD

**Fig. 1:**
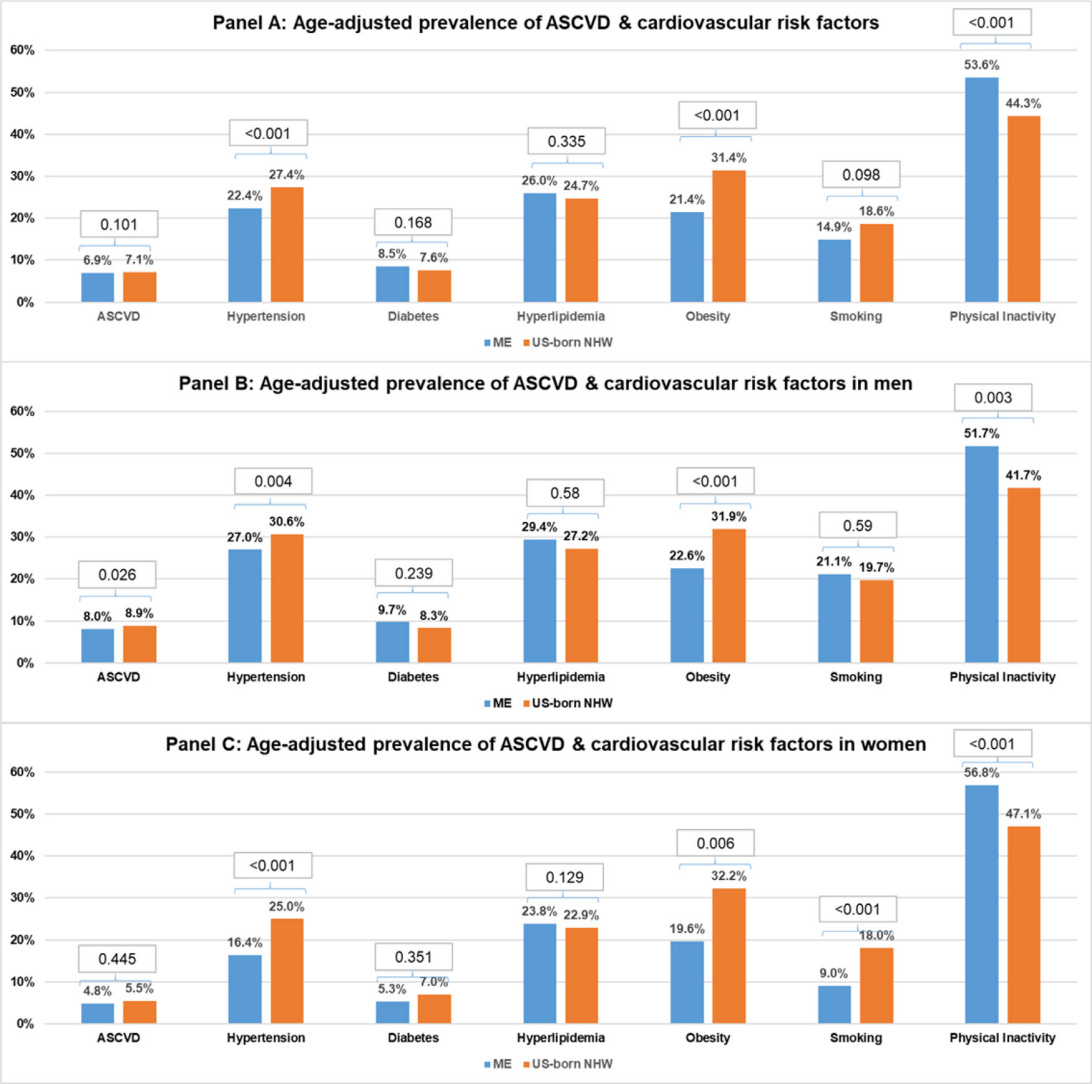
Age-adjusted prevalence of cardiovascular risk factors Panel A: prevalence in total population. Panel B: prevalence in men. Panel C: prevalence in women. Abbreviations: ASCVD, atherosclerotic cardiovascular disease.

The age-adjusted prevalence of cardiovascular risk factors by study group is presented in Fig. 1. Compared to US-born NHW, ME immigrants had lower age-adjusted prevalence of self-reported hypertension (22.4% vs 27.4%, *p* < 0.001) and of obesity (21.4% vs 31.4%, *p* < 0.001). There was no significant difference in the age-adjusted prevalence of diabetes, hyperlipidemia, smoking, and ASCVD, between the 2 groups. The age-adjusted prevalence of insufficient physical activity was however higher among ME immigrants compared with US-born NHW (53.6% vs 44.3%, *p* < 0.001) (Fig 1 Panel A).

When stratified by sex, there was a lower prevalence of hypertension and obesity among ME immigrants for both genders compared with US-born NHW. Additionally, among men, the prevalence of ASCVD was lower among ME immigrants compared to US-born NHW. However, the prevalence of smoking was slightly higher among ME men compared to US-born NHW men. In contast, ME women had lower prevalence of smoking than US-born NHW women (Fig. 1 Panels B & C).

### 3.4 Associations between me origin, cardiovascular risk factors, and ASCVD

In univariable regression analyses, ME immigrants had lower odds of reporting ASCVD and most cardiovascular risk factors than US-born NHWs, although these odds ratios (ORs) were only statistically significant for hypertension (OR 0.72, 95% CI [0.58, 0.89]); and obesity (OR 0.64, 95% CI [0.54, 0.75]). ME immigrants had higher odds of insufficient physical activity (OR 1.29, 95% CI [1.12, 1.49]) compared to US-born NHW. Additionally, MEs had lower odds of having a suboptimal CRF profile (OR 0.73, 95% CI [0.64, 0.82]); with this association remaining unchanged even among participants without ASCVD when compared to US-born NHWs (Table 2).

**Table 2:** Logistic regression for cardiovascular risk factors in ME individuals compared to US-born NHW.

	Model 1*			Model 2†			Model 3‡	
	OR (95% CI)	p-value		OR (95% CI)	p-value		OR (95% CI)	p-value
Hypertension	0.72 (0.58, 0.89)	0.003		0.79 (0.68, 0.91)	0.001		0.71 (0.61, 0.83)	<0.001
Diabetes mellitus	0.87 (0.66, 1.14)	0.316		1.00 (0.81, 1.24)	0.973		0.86 (0.69, 1.06)	0.158
Hyperlipidemia	0.95 (0.78, 1.16)	0.604		1.09 (0.96, 1.25)	0.19		1.07 (0.94, 1.21)	0.301
Obesity	0.64 (0.54, 0.75)	<0.001		0.64 (0.55, 0.76)	<0.001		0.61 (0.52, 0.72)	<0.001
Smoking	0.81 (0.61, 1.08)	0.149		0.78 (0.59, 1.01)	0.062		0.61 (0.46, 0.80)	<0.001
Insufficient physical activity	1.29 (1.12, 1.49)	0.001		1.40 (1.21, 1.63)	<0.001		1.30 (1.11, 1.53)	0.002
ASCVD	0.71 (0.50, 1.01)	0.057		0.84 (0.58, 1.20)	0.32		0.69 (0.45, 1.08)	0.103
Sub-Optimal CRF Profile§	0.73 (0.64, 0.82)	<0.001		0.79 (0.66, 0.94)	0.007		0.71 (0.60, 0.84)	<0.001
Sub-Optimal CRF Profile among those without ASCVD	0.74 (0.67, 0.83)	<0.001		0.79 (0.68, 0.93)	0.004		0.73 (0.61, 0.86)	<0.001

⁎ Unadjusted model.

† Model adjusted for age & sex.

‡ Model 3: Model 2 + income, insurance, education & having a usual place of care.

§ Having 2 or more risk factors Abbreviations: ME, Middle Eastern; US, United States; NHW, non-Hispanic white; OR, Odds Ratios; CI, confidence interval; ASCVD, atherosclerotic cardiovascular disease; CRF, Cardiovascular risk factor profile.

After progressive adjustment for potential confounders (age and sex in model 2, in addition to sociodemographic factors in model 3) all associations remained largely unchanged in both direction and magnitude.

### 3.5 Role of length of stay and other social determinants of health among me immigrants

**Fig. 2:**
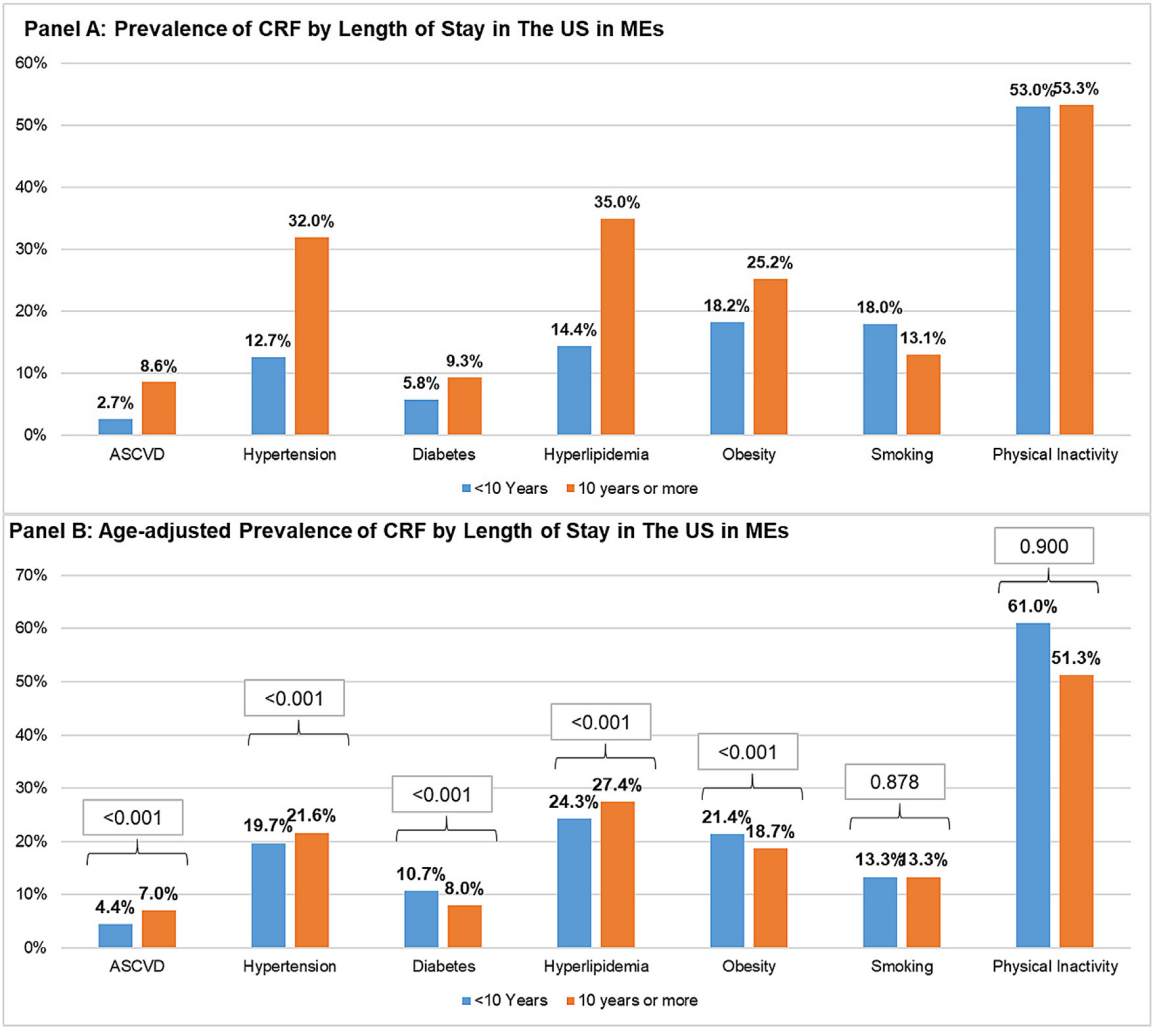
Prevalence of CRF by length of stay in the US Panel A: crude prevalence. Panel B: age-adjusted prevalence. Abbreviations: CRF, cardiovascular risk factors.

The crude and age-adjusted prevalence of cardiovascular risk factors by the length of stay in the US among MEs is presented in Fig. 2. ME immigrants who stayed in the US for 10 years or more had a higher age-adjusted prevalence of ASCVD, hypertension, and hyperlipidemia, and lower prevalence of diabetes, obesity, and insufficient physical activity compared with those who lived in the US for less than 10 years (Fig. 2 Panel B).

**Fig. 3:**
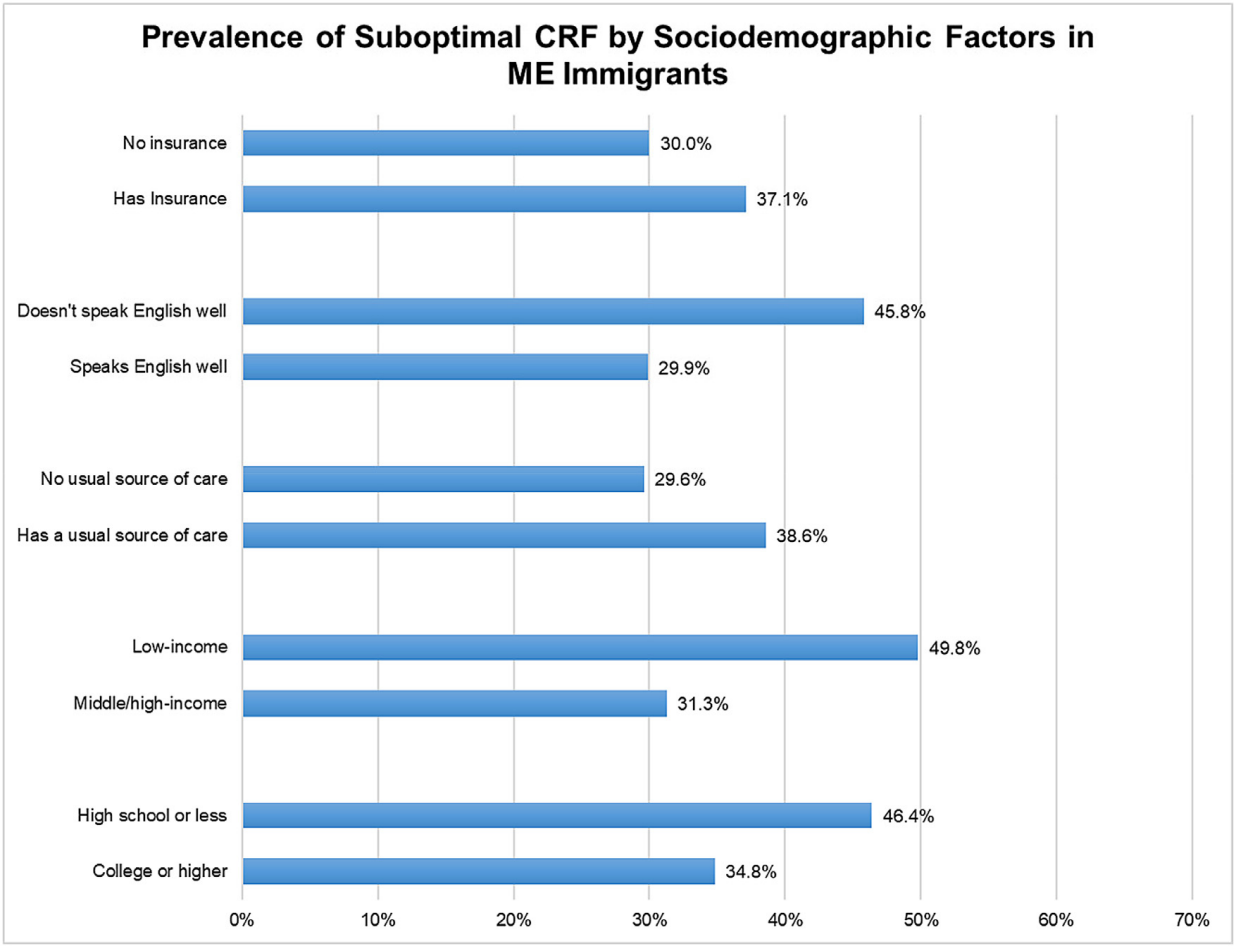
Prevalence of suboptimal CRF by sociodemographic factors in ME immigrants.

The crude prevalence of a suboptimal CRF in ME immigrants was higher within certain subgroups. When comparing the prevalence of suboptimal CRF profile by sociodemographic characteristics among ME immigrants, those with insurance, higher education, have a usual source of care, from low-income households, and with low English language proficiency reported a higher prevalence of a suboptimal CRF (Fig. 3). The sociodemographic factors most strongly associated with reporting a suboptimal CRF included male sex, lower education attainment, and low income (Table 3). Not having a usual source of care was associated with lower odds of reporting a suboptimal CRF. Although low English proficiency was a driver of having a suboptimal CRF, in multivariable analysis, it was associated with lower odds of a suboptimal CRF profile.

**Table 3:** Odds ratios of having a suboptimal CRF in ME immigrants by sociodemographic factors.

	Model 1*		Model 2†	
Age	OR (95% CI)	P-value	OR (95% CI)	P-value
18–44	Reference		Reference	
45–64	2.65 (1.90, 3.69)	<0.001	3.22 (3.05, 3.41)	<0.001
65 and older	12.47 (9.04, 16.92)	<0.001	6.10 (5.69, 6.55)	<0.001
Sex				
Female	Reference		Reference	
Male	1.66 (1.27, 2.15)	<0.001	1.11 (1.08, 1.14)	<0.001
Education				
Some College or Higher	Reference		Reference	
High school or Less	2.40 (1.84, 3.12)	<0.001	1.82 (1.74, 1.94)	<0.001
Income				
Middle/High-Income	Reference		Reference	
Low-Income	1.76 (1.27, 2.45)	0.001	1.74 (1.69, 1.78)	<0.001
Insurance				
Has insurance	Reference		Reference	
No insurance	0.74 (0.48, 1.11)	0.148	1.14 (1.07, 1.21)	<0.001
Usual source of care				
Yes	Reference		Reference	
No	0.39 (0.27, 0.58)	<0.001	0.68 (0.65, 0.72)	<0.001
English Proficiency				
Speaks well	Reference		Reference	
Does not speak well	2.40 (1.78, 3.23)	<0.001	0.76 (0.71, 0.81)	<0.001

⁎ Unadjusted model.

† Model adjusted for all other sociodemographic factors in the table.

## 4 Discussion

In a study using 2012–2018 data from NHIS, we found that ME immigrants in the US seem to have lower prevalence and odds of hypertension and obesity, and of having a suboptimal CRF profilecompared to US-born NHWs. Our results show that ME individuals had a significantly lower prevalence of hypertension and obesity, and lower odds of having a suboptimal CRF compared to US-born NHWs. Despite a lower prevalence of a suboptimal CRF in ME immigrants than in US-born NHWs, we did not find a significant association for ASCVD, although our study may have been underpowered to detect such differences. Of note, ME immigrants staying in the US for 10 years or more had a higher age-adjusted prevalence of hypertension and hyperlipidemia and lower for diabetes, obesity, and insufficient physical activity. Finally, the factors independently associated with higher odds of suboptimal CRF among ME immigrants were male sex, low household income, low English proficiency, and low educational attainment. Not having a usual source of care was associated with lower odds of having a suboptimal CRF in ME immigrants.

Our findings build on the prior published literature in several ways. A previous NHIS analysis using data up to 2016 showed similar prevalence of hypertension and diabetes in ME immigrants in comparison to other immigrant groups in the US [2]. The current manuscript adds to the literature by providing results from more recent years, and examining other relevant cardiovascular risk factors including hyperlipidemia, insufficient physical activity, and smoking; as well as ASCVD.

Our findings, which suggest that ME in the US are a relatively cardiovascular-healthy population, could be explained by several mechanisms. A healthier diet could play a role i.e. Mediterranean diet [17], although the adherence of said diet has not been studied in ME countries or ME immigrants in the US [18]. A second potential mechanism could involve the selection of healthy individuals immigrating into the US, a mechanism that has been described in other groups and limits generalizability of findings from immigrant studies to the countries of origin. Third, oversampling of more educated ME immigrants, and under-detection and/or under-reporting of cardiovascular risk factors in ME immigrants living in the US are also possibilities. The latter has also been proposed for other immigrant populations whose burden of risk factors and ASCVD in NHIS was remarkably lower compared to non-survey studies in the US, such as South Asians [19]. This is important in light of the lower proportion of ME being insured or having a usual source of care observed in our analysis, which is likely to have affected access to healthcare and potentially result in an under-detection of some prevalent risk factors. Studies using objective measures of risk factors in a sample representative of the whole ME population in the US would likely address these potential issues.

Still, important risk factors among ME immigrants in the US noted in our study included insufficient physical activity, hyperlipidemia, and diabetes among men. The burden of insufficient physical activity was particularly high in ME immigrants (53.6% in our analysis); a comparable prevalence (49.2%) was noted in a 2020 meta-analysis of 125 studies conducted in 20 different ME countries [20]. The study identified several barriers to insufficient physical activity including physical environment factors (lack of parks, neighborhood aesthetics, hot arid climate), psychological and social factors (unperceived benefits of physical activity, absence of social support from friends and peers), and conservative social norms particularly relevant for women [20].

Another important finding in our study is that not having a usual source of care was associated with lower odds or reporting a suboptimal CRF profile. This finding is consistent with a study by Samari et al. demonstrating that ME immigrants had significantly lower odds of accessing and utilizing healthcare services compared to US-born NHWs [1]. This, in turn, supports the notion that cardiovascular risk factors may be under-detected in ME immigrants. Further supporting this hypothesis, local studies in the Middle East have noted a high burden of coronary heart disease and its risk factors, however, well-designed population-based studies are lacking in the region.

We also noted the effects of length of stay in the US on ME immigrants. Those who have been living in the US for 10 years or more reported higher age-adjusted prevalence of hypertension, hyperlipidemia, and ASCVD. This finding compiles to a study from the NHIS in 2007 that immigrants in the US (not limited to ME) who stayed in the US for 15 years or more had higher odds of obesity, smoking, and hyperlipidemia, and no increased odds of diabetes or hypertension when compared to those staying less than 10 years [6]. While our findings for hypertension and hyperlipidemia could be explained by better access to screening over time once in the US, this is unlikely to explain the results for ASCVD.

The implications of our study include adding recent, nation-level information about the CRF profile of ME immigrants; the rapidly growing ME immigrant population in the US may benefit from being highlighted in future research. A better understanding of their cardiovascular health may inform health policies to improving their and immigrant health in general. Further, assistance and awareness programs may be directed to especially vulnerable individuals with adverse sociodemographic factors who remain with undiagnosed conditions.

### 4.1 Study limitations

The results of our study need to be viewed considering a few limitations. First, NHIS data can only identify ME participants that were born in the Middle East by virtue of the question about country of birth. Currently, the NHIS does not have a way to identify individuals of ME origin who were born in the US or other regions, therefore ME immigrants in the US may be under-detected in our sampleIn addition, NHIS only provides the region of birth for participants without information on the specific countries of birth. Therefore, there is no way to identify which specific ME countries are more represented, and if our results are skewed to certain countries of origin. Second, NHIS is based on self-report, which has the potential of introducing information bias, especially in the setting of language barriers. However, the use of highly trained field personnel and where appropriate, interpreters in situations of low English proficiency, ensures the collection of high-quality consistent data. The NHIS is a reliable source of national health statistics in the US and has been used to study other immigrant populations in the US. [2] However, our group has previously highlighted important potential limitations of a survey like NHIS in immigrant populations [19]. Third, although we compiled several years as possible of NHIS data, our sample of ME participants was relatively small, and some of the comparisons may have been underpowered to detect statistically significant differences between the study groups (e.g., diabetes among men, ASCVD overall). Fourth, the cross-sectional nature of the study does not establish causality. Finally, even after adjusting for confounders (due to nature of the study), residual confounding is still a possibility.

## 5 Conclusions

ME immigrants in the US seem to exert overall lower odds of hypertension, obesity, and of having a suboptimal CRF profile compared to US-born NHWs. Still, our analysis identified very high levels of insufficient physical activity in this group, as well as a significant burden of hyperlipidemia and diabetes. These findings could be used to inform culturally targeted interventions to help optimize their cardiovascular risk. More studies are needed to better characterize the cardiovascular risk of ME immigrants living in the US, as well as the health effects of length of stay in the country.

## Declaration of Competing Interest

Dr. Nasir is on the advisory board of Amgen, Novartis, Medicine Company, and his research is partly supported by the Jerold B. Katz Academy of Translational Research. No other conflicts of interest relevant to the content of this manuscript were reported by the authors.

## Appendix Supplementary materials

Image, application 1
