# Supplementary material for: Cardiovascular risk profile of Middle Eastern immigrants living in the United States-the National Health Interview Survey
Source: Am J Prev Cardiol. 2021 Dec 27;9:100312. doi: 10.1016/j.ajpc.2021.100312 (PMC8732795; doi:10.1016/j.ajpc.2021.100312)
Supplement: Supplementary file 1 [file mmc1.docx]

# Supplementary Tables

## Supplementary Table 1. Question items used to define cardiovascular risk factors in NHIS

| Risk factor | NHIS item |
| --- | --- |
| Hypertension | - “Have you ever been told by a doctor or other health professional that you had hypertension, also called high blood pressure?” |
| Diabetes | - “Have you ever been told by a doctor or other health professional that you have diabetes or sugar diabetes?” |
| Hyperlipidemia | - “Have you ever been told by a doctor or other health professional that you had high cholesterol?” |
| Obesity | - Calculated using self-reported weight and height   - “How much do you weigh without shoes?”   - “How tall are you without shoes?” |
| Smoking | - “Current smoking status” |
| Insufficient physical activity | - Variable created based on questions regarding the frequency and intensity of physical activity. Insufficient physical activity defined as not participating in >150 minutes per week of moderate-intensity aerobic physical activity, >75 minutes per week of vigorous-intensity aerobic physical activity, or a total combination of ≥150 minutes per week of moderate/vigorous-intensity aerobic physical activity |

Abbreviations: NHIS, National Health Interview Survey
